# Single exposure to near-threshold 5G millimeter wave modifies restraint stress responses in rats

**DOI:** 10.1265/ehpm.24-00321

**Published:** 2025-05-03

**Authors:** Akiko Matsumoto, Ikumi Endo, Etsuko Ijima, Akimasa Hirata, Sachiko Kodera, Masayoshi Ichiba, Mikiko Tokiya, Takashi Hikage, Hiroshi Masuda

**Affiliations:** 1 Department of Social and Environmental Medicine, Saga University School of Medicine, 5-1-1 Nabeshima, Saga 849-8501, Japan; 2 Department of Environmental Medicine, Kurume University School of Medicine, Kurume 830-0011, Japan; 3 Department of Electrical and Mechanical Engineering, Nagoya Institute of Technology, Nagoya 466-8555, Japan; 4 Faculty of Information Science and Technology, Hokkaido University, Sapporo 060-0814, Japan

**Keywords:** Quasi-millimeter waves, 5G, Whole-body exposure, Stress response biomarkers, Temperature

## Abstract

**Background:**

In response to growing concerns about the health effects of quasi-millimeter waves (qMMW) used in 5th-generation wireless systems, conservative whole-body exposure thresholds based on indirect evidence have been proposed. The guidelines define a whole-body average specific absorption rate (WBA-SAR) of 4 W/kg which causes a 1 °C increase in core temperature, as the operational threshold for adverse health effects. To address the lack of direct evidence, we recently reported that a 30-minute exposure to qMMW at 4.6 W/kg resulted in a 1 °C increase in rat core temperature. Here, we further analyzed the near-threshold stress response for the first time, using biological samples from the aforementioned and additional experiments.

**Methods:**

A total of 59 young Sprague-Dawley rats (240–322 g) were exposed to 28 GHz for 40 minutes at WBA-SARs of 0, 3.7, and 7.2 W/kg, under normal (22.5 °C, 45–55% humidity), and heat (32 °C, 70% humidity) conditions. Rats were restrained in acrylic holders for dose control. We repeatedly measured serum and urinary biomarkers of stress response, aggregated the data, and analyzed them using a single statistical mixed model to subtract the effects of sham exposure and between-subject variation.

**Results:**

Sham exposure induced stress responses, suggesting an effect of restraint. After the subtraction of the sham exposure effect, 28 GHz appeared to induce stress responses as evidenced by elevated serum-free corticosterone 1 or 3 days after the exposure, which was more evident in animals with a change in rectal temperature exceeding 1 °C. Urinary-free catecholamines demonstrated an inhibitory property of 28 GHz frequency exposure on the stress response as evidenced by noradrenaline on the day of exposure. Heat exposure enhanced this effect, suggesting a possible role of noradrenaline in heat dissipation by promoting cutaneous blood flow, a notion supported by the correlation between noradrenaline levels and tail surface temperature, a critical organ for heat dissipation.

**Conclusions:**

This study is the first to demonstrate that qMMW whole-body exposure can alter the stress response as indicated by corticosterone and noradrenaline at near-threshold levels. Our findings may provide insight into the biological basis of the whole-body exposure thresholds in the international guidelines.

**Supplementary information:**

The online version contains supplementary material available at https://doi.org/10.1265/ehpm.24-00321.

## 1 Background

In recent years, wireless communication devices have become commonplace with the spread of smartphones. On the other hand, opportunities to be exposed to radiofrequency have inevitably increased in our daily lives, raising concerns about the potential adverse health effects of exposure. According to the WHO, there is no clear evidence of adverse health effects from radiofrequency at levels below current international guidelines. The International Commission on Non-Ionizing Radiation Protection (ICNIRP) guidelines for limiting exposure to radiofrequency radiation (100 kHz and 300 GHz) prescribed a dose limit of 0.4 W/kg whole-body averaged specific absorption rate (WBA-SAR), derived from the operational adverse health effect threshold of 4 W/kg from available evidence and a reduction factor of 10 [1].

**Fig. 1:**
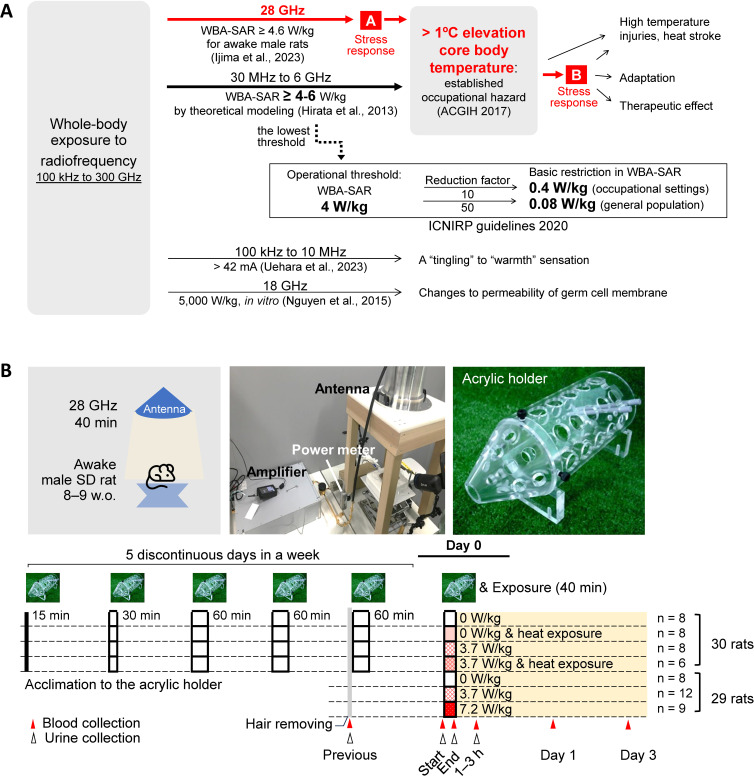
Study positioning and protocols. (A) Study positioning: The reasonably expected physical effect of the lowest dose of radiofrequency is temperature elevation. The ICNIRP guidelines (2020) covering the 100 kHz to 300 GHz range adopted a calculated estimate of the equivalent dose of a 1 °C increase in core body temperature, the established occupational hazard. From the calculated result, WBA-SARs of 4–6 W/kg from 30 MHz to 6 GHz radiofrequency at an ambient temperature of 28 °C for the naked and resting human body, 4 W/kg was adopted as the operational threshold. Our study series, the first exposure experiment with accurate dosimetry for whole-body exposure at 28 GHz (Ijima et al., 2023 and current study) at near-threshold levels, confirmed 4.6 W/kg as the equivalent dose for a 1 °C increase in core body temperature in rats after a 30 min exposure. In the present study, indicated by red arrows and fonts, we have attempted to characterize the stress response (homeostatic regulation) expected to occur in A and B. (B) Study design: Thirty rats were acclimated to the acrylic holders that were used to ensure 28 GHz exposure for 5 discontinuous days in a week before exposure (15–60 minutes (min) per day); the other 29 rats were not acclimated. The dorsal skin hair was removed 1–3 days before the experiment under 2% isoflurane anesthesia. Blood samples were collected from the jugular vein under 2% isoflurane anesthesia at 1–3 days before exposure (previous), immediately before exposure (start), immediately after exposure (end), and 1–3 h after exposure (1–3 h). In addition to blood samples, we collected urine samples that had leaked into the anesthetic box (acrylic plate). Urine specimens were often missing, due to the difficulty in controlling urination in animals. The rats were maintained in acrylic holders under 2% isoflurane anesthesia before 28 GHz exposure and fitted with optical fiber probes for measurements of back skin (dermal), tail skin (tail), and rectum (rectal) temperature, and dermal and tail blood flow. After awakening from temporary anesthesia, rats were exposed to 28 GHz frequency for 40 min. The laboratory was configured to operate under two distinct environmental conditions: 1) an ambient temperature of approximately 22.5 °C, with a 45–55% humidity level; and 2) an ambient temperature of approximately 32 °C, with an approximately 70% humidity level (heat exposure). Anesthesia was usually performed in the normal environment, and a heater, at 37 °C, was placed under the animals to maintain the body temperature during anesthesia. 28 GHz exposure was performed at WBA-SARs of 0, 3.7, or 7.2 W/kg. From the perspective of animal welfare, 7.2 W/kg treatment was applied only under the normal environment, because it promotes an increase in rectal temperature by approximately 2 °C. WBA-SAR: whole-body average specific absorption rate; w.o.: week-old; min: minutes.

Wireless communication sources are shifting to 5th generation wireless communication systems (5G), including the quasi-millimeter wave in the range of 24 to 28 GHz (qMMW) [2], to improve the quality of service by increasing data transmission speeds and reducing latency. As summarized in Fig. 1A, the current ICNIRP guidelines published in 2020 include the qMMW range [1], and the same operational threshold of 4 W/kg WBA-SAR has been applied to qMMW as to the lower frequencies used for pre-5G (conventional wireless communication systems), for several key reasons. First, of the three known health effects of radiofrequency exposure, “nerve stimulation,” observed at frequencies ≤10 MHz [3], is not expected to occur above 6 GHz because the majority of energy at these higher frequencies is absorbed in the dermal layers [4, 5]. Second, while “changes in cell membrane permeability” have been observed in bacterial membranes after exposure to 18 GHz radiofrequency, these effects are only seen following an increase in temperature [6]. Finally, a “temperature rise” in the core of the body via blood circulation is to be expected, as is known from experience with infrared radiation (>300 GHz) [7] and from scientific evidence for lower frequencies <6 GHz [8]; a temperature rise of 1 °C, an established occupational hazard [9], is to be expected at the lowest intensity of all frequency bands so far.

The operational threshold for the ICNIRP guidelines was derived from an estimated relationship between 30 MHz to 6 GHz radiofrequency intensity and body temperature rise using human body models [8], and thermophysiological data reported from 2001 to 2005 on 100 MHz to 2.5 GHz radiofrequency exposure in volunteers [10–12]. The computationally estimated WBA-SARs of 4–6 W/kg (conservatively 4 W/kg) were thus used as the basis for the ICNIRP guidelines [1]. However, the ICNIRP listed the lack of data in the Gaps of Knowledge document for future guidelines development, including on core temperature rise and health effects [13]. Because the above frequencies were lower than the qMMW, we recently attempted to address the lack of evidence; the equivalent intensity for the 28 GHz (qMMW) was reported to be WBA-SAR of 4.6 W/kg to cause a 1 °C increase in rat core body temperature after a 30-minute (min) exposure [14]. Here, we further evaluated stress response markers to provide, for the first time, multiple lines of evidence other than body temperature from the near-threshold exposure.

Stress response signals detected by glucocorticoid and catecholamine in biological samples are widely used to quantify the impacts of environmental factors [15–19]. Stress, which requires physical changes to maintain homeostasis, is sensed by peripheral receptors for various physical stimuli such as heat and mechanical pressure [15, 20]. The signals are integrated into the hypothalamus, resulting in a stress response via the sympathetic nerve and the hypothalamic-pituitary-adrenal axis, which promote the release of catecholamines and glucocorticoids to achieve necessary adaptation [19, 21]. Biomarkers of oxidative stress are also valuable biomarkers for stress response [22, 23], including thermal stress [24]. The generation of superoxide anions (O_2_−), hydrogen peroxide (H_2_O_2_), and hydroxyl radicals (•OH) throughout mitochondrial ATP synthesis are considered to initiate the oxidative burden [25, 26], and surplus amounts of these compounds are known to cause the aggregation of pathogenic substances, such as lipid peroxide and impaired DNA; however, fundamentally these compounds represent a favorable stimulus for safeguarding cellular homeostasis [27, 28]. Exploring the limits of radiofrequency intensity that require homeostatic adjustments helps verify the current operational thresholds for radiofrequency exposure.

To meet this expectation, biological samples collected in the reported animal experiments [14] (29 rats) and in additional experiments (30 rats) were analyzed. With repeated observation points on the stress response, we created a comprehensive database that included experimental settings such as acclimation regimen, timing of sample collection, thermophysiological parameters, and other relevant factors, and analyzed in a single statistical model that maximized statistical power and allowed us to subtract the effects of sham exposure and control for between-subject variation.

## 2 Methods

### 2.1 Animals

All experiments in this study followed the regulations set forth by the Committee on Animal Experiments at Kurume University School of Medicine (2020-174 and 2021-150). Male Sprague-Dawley rats, aged 8–9 weeks, were procured from Japan SLC Inc., Shizuoka, Japan, and were housed in a sterile environment with a regulated temperature of 22.5 ± 1 °C, humidity level of 50 ± 20%, and 12/12 h light/dark cycle, with access to food and water ad libitum. The dorsal skin hair of the rats was removed 1–3 days before the experiment, under 2% isoflurane anesthesia, using dog grooming clippers (Model ER803P, Panasonic Corp, Kadoma, Japan).

#### 2.1.1 Exposure experiments

Protocols are summarized in Fig. 1B, and the part for 29 rats was described in our previous study [27]. Briefly, the 28 GHz continuous wave output from a signal generator (JOGSAG1401, SAF Tehnika, Riga, Latvia) was administered to the awake rats through a horn antenna equipped with a dielectric lens, via an amplifier (AMP6034, Exodus Advanced Communications, Las Vegas, Nevada). The effective irradiated area on the back of a rats, which was higher than 1/*e* of the maximum power density, was 318.4 cm^2^.

### 2.2 Analysis of free corticosterone in the serum

Modifying our extraction method for human urinary cortisol and cortisone measurements [29], samples were pretreated with solid phase extraction. Briefly, serum samples stored at −80 °C were thawed immediately before pretreatment, and 20% of 1 M phosphate buffer (pH 7) pre-mixed with an internal standard (corticosterone-9,11,12,12-d4, FUJIFILM Wako Pure Chemical Corporation, Osaka, Japan) was added to 20 µL of serum aliquots. The extraction was performed using a MonoSpin C18 column (GL Science, Tokyo, Japan), a centrifugal solid-phase extraction column; the column conditioning was performed by loading it with 300 µL of methanol and centrifuging it, followed by loading with 300 µL of 10 mM phosphate buffer (pH 7) and centrifugation at 2,300 × *g* for 1 min. The serum samples mixed with the internal standard were placed on the column and allowed to stand for 1 h at 20–25 °C. After centrifugation at 2,300 × *g* for 2 min to remove non-adsorbed material, the column was washed sequentially with 600 µL of liquid chromatography-mass spectrometry (LC-MS) grade pure water (Merck Millipore, Burlington, Massachusetts) and 300 µL of acetate buffer (18 mM, pH 5.3, LC-MS grade, Wako Pure Chemical Corporation, Osaka, Japan). The target material was eluted with 100 µL of methanol (LC-MS grade, Sigma-Aldrich, St Louis, MO), centrifugally concentrated, and redissolved in 60 µL of methanol:acetate (40:60, v:v) buffer (18 mM, pH 5.3, LC-MS grade) mixture. Chromatography was performed using an LC system (Shimadzu Corporation, Kyoto, Japan) consisting of an autosampler (SIL-30AC), binary pump (LC-30AD), column oven (CTO-20AC), and mass spectrometer (LCMS-8030). The separation column was an Ascentis Express C18 column (length: 100 mm, inner diameter: 2.1 mm, particle diameter: 2.7 µm; Sigma-Aldrich, St Louis, MO). The autosampler and the column temperatures were set at 4 °C and 50 °C, respectively. Gradient separation was conducted with 18 mM acetic acid buffer (pH 5.3) (A) and methanol (B) as follows: 0–2 min, 40–52% B; 2–5 min, 52% B; 5–6 min, 52–100% B; 6–7 min, 100% B; 7–7.1 min, 40–100% B; 7.1–9.5 min, 40% B. Flow rate and injection volume were set at 0.5 mL/min and 10 µL, respectively. The mass-to-charge ratio (*m*/*z*) for corticosterone and corticosterone-D4 were 347.30>120.90 and 351.20>120.90, respectively. The optimization function of the LabSolutions software for LC-MS (Shimadzu) was used to fine-tune *m*/*z* and voltage values.

### 2.3 Analysis of free catecholamines in the urine

Measurements were made using an automated catecholamine analyzer HLC^®^-725CA III (Tosoh Corporation, Tokyo, Japan). This instrument derivatizes catecholamines with diphenylethylenediamine, separates and purifies them through two-step column switching, and analyzes them using fluorescence. According to the manufacturer, the quantitative ranges for noradrenaline, adrenaline, and dopamine were 0.017–0.676 ng/mL, 0.018–0.732 ng/mL, and 0.015–0.612 ng/mL, respectively (100 ± 20% accuracy). Rat urine was diluted ≥250-fold in an acidic diluent (0.1 M ascorbic acid with 0.1 M EDTA-2Na in 2% perchloric acid) before analysis, and 0.2 mL of the diluted samples were injected into the system.

### 2.4 Urinary creatinine measurement

Frozen urine samples were thawed immediately before assessment, gently stirred, and diluted 20–200 times with 50% methanol. A high-performance liquid chromatography (HPLC) system (Shimadzu Corporation) consisting of an autosampler (SIC-20A HT), binary pump (LC-20AD), column oven (CTO-10AC), and UV spectrophotometer (SPD-10A) was used for this analysis. The separation was performed using an ODS column, Shim-pack XR-ODS II (length: 75 mm, inner diameter: 3 mm, particle size: 2.2 µm; Shimadzu), and the temperature of the column oven was adjusted to 50 °C. Gradient separation was conducted using 20 mM KH_2_PO_4_, 3 mM sodium decanesulfonate (pH 3.3) (C), and acetonitrile (D; concentration: 8–15%). Flow rate and injection volume were set at 1 mL/min and 4 µL, respectively. Absorbance was measured at a wavelength of 225 nm.

### 2.5 Measurement of oxidative stress markers in the serum

Serum diacron reactive oxygen metabolites (d-ROMs) and biological antioxidant potential (BAP) were measured using FREE Carrio Duo (Diacron International s.r.l., Italy, Wismerll Co., Ltd., Tokyo, Japan), following the manufacturer’s instructions. Serum d-ROM levels indicate the total amount of peroxides in U.CARR, where a unit is equivalent to approximately 0.08 mg H_2_O_2_/dL, including the metabolites produced when reactive oxygen species oxidize lipids, proteins, amino acids, and nucleic acids; and is quantified colorimetrically using the color reaction caused by the oxidation of N,N-diethyl-1,4-benzenediamine in the presence of iron ions in a pH 4.8 acetate buffer. BAP colorimetrically quantifies the color fade of Fe^3+^ ion (FeCl_3_-thiocyanate coloration) by reducing compounds such as glutathione, thiols, ascorbic acid, serum proteins, bilirubin, and uric acid in the serum (in µmol/L). According to the manufacturer of FREE Carrio Duo, the quantification ranges for d-ROMs and BAP are 40–1000 U.CARR and 500–6000 µmol/L, respectively. The respective coefficients of variation for intra- and inter-day reproducibility were as follows: 2.07% (20 repeated measures at 300 U.CARR) and 1.79% (20 repeated measures at 295 U.CARR) for d-ROMs; and 2.15% (20 repeated measures at 2266 µmol/L) and 3.05% (20 repeated measures at 2246 µmol/L) for BAP. The d-ROMs and BAP values were not assessed for samples with obvious hemolysis or insufficient serum volume, while samples with only half the volume were measured at half volume, and then doubled.

### 2.6 Statistical analysis

#### 2.6.1 Databases

The distribution of subjects is shown in Table 1. We pooled the databases of two cohorts, one with acclimation and one without. To account for the difference in procedure, the covariate of acclimatization was applied to the statistical models (see 2.6.4).

**Table 1:** Distribution of observations and animals.

	**Corticosterone**					**Catecholamine**					**d-ROMs and BAP**				
	**Serum**					**Urine**					**Serum**				
Total number	267 observations, 54 rats					188 observations, 59 rats					279 observations, 59 rats				
WBA-SAR, W/kg	0		3.7		7.2	0		3.7		7.2	0		3.7		7.2
Heat exposure	−	+	−	+	−	−	+	−	+	−	−	+	−	+	−
N of animals (with acclimation)	16 (8)	8 (8)	16 (8)	6 (6)	8 (0)	16 (8)	8 (8)	20 (8)	6 (6)	9 (0)	16 (8)	8 (8)	20 (8)	6 (6)	9 (0)
			
N of observations															
Previous	20	5	20	6	7	17	5	18	5	5	20	5	20	6	7
Start	16	8	15	6	8	13	5	16	5	9	16	8	15	6	8
End	15	7	15	6	8	13	6	17	4	9	15	7	15	6	8
1–3 h	14	8	15	6	8	7	6	15	5	8	14	8	15	6	8
Day 1	8	4	7	3	4	–	–	–	–	–	8	4	7	3	4
Day 3	8	4	9	3	4	–	–	–	–	–	8	4	9	3	4

Urine collection on days 1 and 3 was unsuccessful. Previous: 1–3 days before exposure. Start: immediately before exposure. End: immediately after exposure. 1–3 h: 1–3 h after exposure. The room temperature and humidity during the experiment were approximately 22.5 °C and 45–55% (ambient), or 31.5 °C and 70% (heat exposure), respectively. WBA-SAR: whole-body average specific absorption rate.

#### 2.6.2 Main outcomes

The main outcomes were free corticosterone in the serum (ng/mL), free catecholamines (noradrenaline, adrenaline, and dopamine) in the urine (ng/mg creatinine), serum d-ROMs (U.CARR), and serum BAP (µmol/L). Serum corticosterone levels were expressed by true numerical values, as logarithmic transformation failed to improve normality (Fig. S1). The urinary catecholamine, d-ROMs, and BAPs were subjected to logarithmic transformation to approximate a normal distribution (Figs. S2 and S3).

#### 2.6.3 Explanatory variables

The explanatory variables included experimental time course (time) and 28 GHz and heat exposures. The time categories “day 1” and “day 3” were combined into one category (days 1–3) due to the limited number of data points. Time categories (previous, start, end, 1–3 h, and days 1–3) were used to estimate non-frequency and non-heat effects (sham effect), such as restraint by acrylic holders. Since the exposure effect can only be detected afterward, the interaction terms between 28 GHz frequency and time (28 GHz × end, 28 GHz × 1–3 h, and 28 GHz × days 1–3) were used as the main explanatory variables to estimate the effect of 28 GHz. Similarly, the interaction terms between heat exposure and time (heat × end, heat × 1–3 h, and heat × days 1–3) were used to estimate the effect of heat exposure. The interaction terms between 28 GHz, heat exposure, and time (28 GHz × heat × end, 28 GHz × heat × 1–3 h, and 28 GHz × heat × days 1–3) were used to evaluate the synergistic effects of 28 GHz and heat exposure. Body temperature and blood flow were successfully monitored in the majority of rats (n = 50–54/59) and were integrated into the database and used as explanatory variables in secondary analyses.

#### 2.6.4 Covariates

We conducted an initial analysis using samples from animals before the experiments (corresponding “previous”) to identify the following covariates. 1) “Acclimation to acrylic holders” based on the negative and positive association with dopamine, and BAP, respectively (Table S1), which half of the rats correspond to. 2) “Time of sample collection” considering the circadian rhythm. Although a rank correlation between the time of sample collection and biomarker levels was not detected (p > 0.16, Table S1), an elevated urinary catecholamine level was observed after 14:00 in a 3-group comparison (Fig. S4). Therefore, a two-category variable was created to represent the collection time (before or after 14:00) as a covariate.

#### 2.6.5 Mixed model incorporating repeated measurements

The effect of 28 GHz and heat exposure on biomarkers was estimated using a mixed model (PROC MIXED in SAS 9.4 TS Level 1M5 for Windows, SAS Institute, Cary, NC, USA), which accounted for repeated measurements, individual variability, and the timing of rat delivery (15 categories between March and August 2021) as random effects. Fixed effects were as mentioned above. Initially, we assumed a linear dose-response for the association between 28 GHz frequency and the biomarkers, i.e., 28 GHz exposure was used as a rank variable (0 W/kg = 0, 3.7 W/kg = 1, and 7.2 W/kg = 2). Subsequently, for items flagged by a significant association, further estimation without assuming a linear dose-response was performed and visualized, by employing 28 GHz as a class variable (PROC MIXED using the LSMEANS statement).

#### 2.6.6 Nonparametric tests for relationships between two variables

Spearman’s rank correlation coefficients (ρ) were computed to examine the non-linear association between two continuous variables, biomarkers and physiological indicators (body temperatures and blood flow), or between rank variables (acclimation and time of sample collection), and continuous variables (PROC CORR in SAS 9.4). Covariates were indicated in each table.

#### 2.6.7 Association between body temperature and biomarker levels

**Fig. 2:**
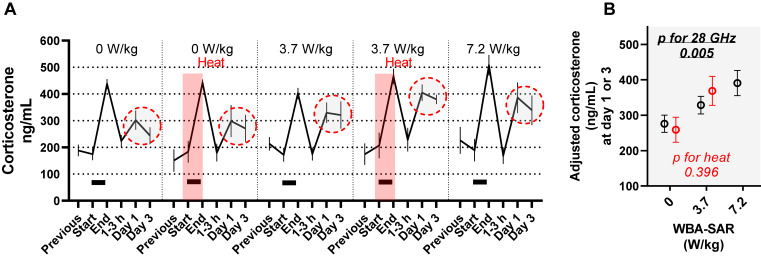
Effect of 28 GHz and/or heat exposure on free corticosterone levels in serum. (A) Means and standard errors (SEMs). Black bars indicate 28 GHz exposure. Red shading indicates exposure to heat at a temperature of 31.5 °C and humidity of 70%. The red dashed circle corresponds to the data extracted in (B). The data points enclosed by the red dashed line (days 1–3) are the source data for the adjusted values plotted in Fig. 2B. (B) Extracted multi-adjusted mean and SEMs. According to a mixed model estimation, 28 GHz affected “Day 1” and “Day 3” (day 1–3) levels of corticosterone (see Table S2). The multi-adjusted means and SEMs corresponding to “Day 1” and “Day 3” were plotted to depict the relationship without an assumption of linear correlation. The least squares means and SEMs based on exposure conditions (red circles indicate heat) were computed using another mixed model with fixed effects of acclimation procedures performed before exposure experiments, the time of sample collection (before/after 14:00), days after exposure treatment (1 or 3), and the random effect of the time of rat procurement (time of the experiment). WBA-SAR: whole-body average specific absorption rate. ‘Heat’ indicates exposure to a hot and humid environment at the experimental temperature of 31.5 °C and humidity of 70%. P-values were computed with the mixed model in Table S2.

**Fig. 3:**
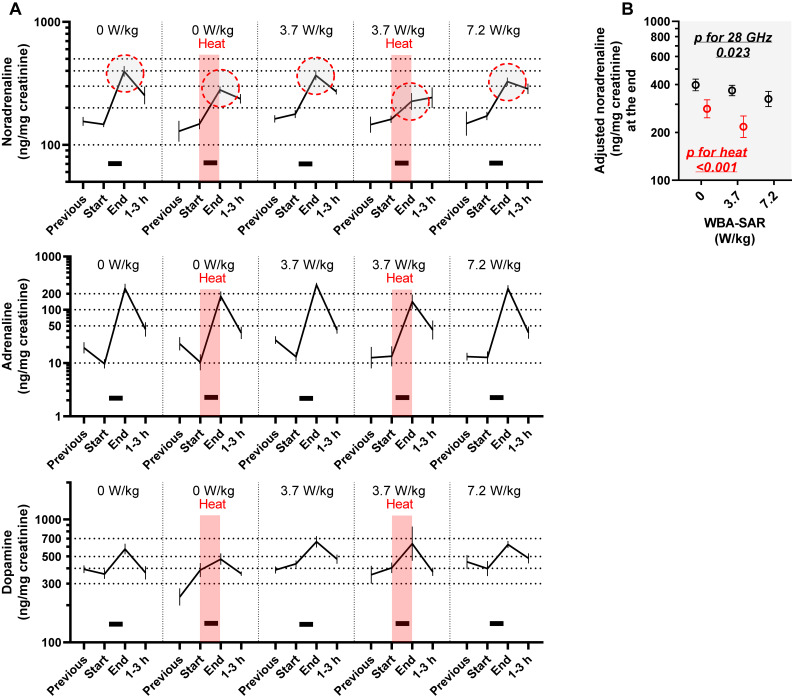
Effect of 28 GHz and/or heat exposure on free catecholamine levels in rat urine. (A) Geometric means and standard errors (SEMs). Black bars indicate 28 GHz exposure. Red shading indicates exposure to heat at a temperature of 31.5 °C and humidity of 70%. The red dashed circle corresponds to the data extracted in (B). The data points enclosed by the red dashed line are the source data for the adjusted values plotted in Fig. 3B. (B) Extracted and multi-adjusted geometric mean and SEMs. According to a mixed model estimation, 28 GHz and heat exposure affected the “End” level of noradrenaline (see Table S4). The multi-adjusted means and SEMs for “End” were plotted to depict the relationship without an assumption of linear correlation. Least squares geometric means and SEMs based on exposure conditions (red circles indicate heat) were computed using another mixed model with fixed effects of acclimation procedures performed before exposure experiments, the time of sample collection (before/after 14:00), and the random effect of the time of rat procurement (time of the experiment). WBA-SAR: whole-body average specific absorption rate. ‘Heat’ indicates exposure to a hot and humid environment at the experimental temperature of 31.5 °C and humidity of 70%. The p-values were computed with the mixed model in Table S4.

**Fig. 4:**
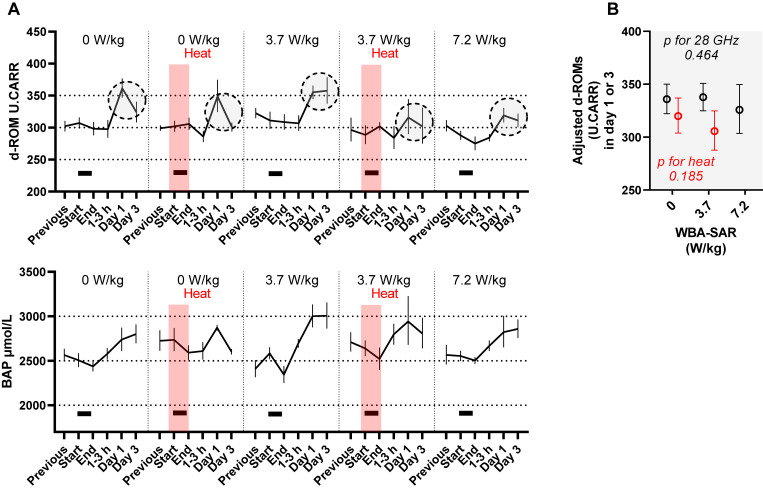
Effect of 28 GHz and/or heat exposure oxidative stress markers in rat serum. (A) Geometric means and standard errors (SEMs). Black bars indicate 28 GHz exposure. Red shading indicates exposure to heat at a temperature of 31.5 °C and humidity of 70%. The data points enclosed by the dashed line (days 1–3) are the source data for the adjusted values plotted in Fig. 4B. (B) Extracted and multi-adjusted geometric mean and SEMs. Although no association was found between oxidative stress markers and 28 GHz or heat exposure (see Table S6), the multi-adjusted means and SEMs corresponding “Day 1” and “Day 3” (day 1–3) were plotted to depict the relationship without an assumption of linear correlation. Least squares geometric means and SEMs based on exposure conditions (red circles indicate heat) were computed using another mixed model with fixed effects of acclimation procedures performed before exposure experiments, the time of sample collection (before/after 14:00), days after exposure treatment (1 or 3), and the random effect of the time of rat procurement (time of the experiment). WBA-SAR: whole-body average specific absorption rate. ‘Heat’ indicates exposure to a hot and humid environment at the experimental temperature of 31.5 °C and humidity of 70%. The p-values were computed with the mixed model in Table S6.

To validate the thermal effects of 28 GHz exposure, we analyzed the relationship between body temperature and biomarkers. We examined rank correlation as mentioned in 2.6.6 for the first step, and then depicted the non-linear relationship for the flagged associations by significance by computing corresponding estimation for quartiles of temperatures (PROC MIXED using the LSMEANS statement). Details of the models are shown in the legend of Figs. 2, 3, and 4.

## 3 Results

### 3.1 Body temperature rise by 28 GHz and/or heat exposure

As depicted in Fig. S5, multivariate adjusted mean of rectal temperature in the sham group increased by 0.5 °C after the experiment (sham effect). 28 GHz and heat exposures increased body temperature dose-dependently, for example, rectal temperature increased by 1.3 °C at WBA-SAR 3.7 W/kg and by 1.9 °C at 7.2 W/kg. An interactive effect between 28 GHz and heat exposure was observed in tail temperatures (rightmost panels in Figs. S5A and S5B, p for interaction = 0.023 and 0.012 for temperature change and end temperature, respectively).

### 3.2 Free corticosterone in the serum

Levels of free corticosterone in 267 serum samples from 54 rats are plotted in Fig. 2A. The levels of corticosterone increased in “end” samples, regardless of and heat exposure (Table S2; partial regression coefficient (β) = 238 for “end”, p < 0.0001) indicating a restraint-induced stress response. A similar but mild elevation was found in the “day 1” and “day 3” samples (Table S2; β = 86 for “1–3 days”, p = 0.001), in which additional effect by 28 GHz was observed (Table S2; β = 55 for “28 GHz × 1–3 days” per one-order increase, p = 0.005), while no additional effect by heat was suggested (Table S2; β = 27 for “heat × 1–3 days”, p = 0.396). To visualize the positive and linear dose effect of 28 GHz in the “day 1” and “day 3” samples, multivariable-adjusted means were plotted in Fig. 2B.

Next, we examined the relationship between corticosterone and body temperature change. We found a strong rank correlation between corticosterone levels (days 1–3) and ΔT, particularly in rectal and dermal ΔT (Table S3; ρ = 0.4, p < 0.01, for both associations). To depict the association, we divided observations into quartiles by ΔT or T-end (q1 to q4) and computed corresponding levels of corticosterone. As shown in Fig. S6, we found a non-linear relationship, with a positive association evident in a range of rectal ΔT above 1 °C; multivariate-adjusted means of corticosterone levels (days 1–3) were 276, 279, 334, and 366 ng/mL for q1, q2, q3, and q4, respectively.

### 3.3 Free catecholamines in the urine

Free catecholamine concentrations in 188 urine samples were measured and plotted (Fig. 4A). Similar to corticosterone, sham effects, elevations at the end of exposure, were detected (Table S4; β = 0.41–3.16 for “end”, p < 0.0001). Among three kinds of catecholamines, noradrenaline elevation was inhibited strongly by heat exposure (Table S4; β = −0.38 for “heat × end”, p = 0.001), and mildly by 28 GHz (Table S4; β = −0.14 for “28 GHz × end”, p = 0.023). The synergistic association (interaction) was not significant (p = 0.45 by a mixed model including the addition of the interactive term, ‘28 GHz exposure × heat × end’, to the model in Table S4). An additive effect of heat and 28 GHz exposure was visualized in Fig. 3B with multivariate adjustment.

Next, we found a strong rank association between tail temperature at the end (T-end) and noradrenaline (end) (Table S5; ρ = −0.46, p = 0.004). To illustrate the association, we estimated noradrenaline (end) corresponding to the quartiles of ΔT or T-end with multivariate adjustment. As shown in Fig. S7, the higher two groups of T-end (q3 and q4) had lower noradrenaline (end) (p for the difference between q2 tail T-end and q3 tail T-end = 0.065, and similarly between q2 and q4 = 0.051).

### 3.4 Oxidative stress indicators

Oxidative stress levels were measured in 279 serum samples from 59 rats and plotted in Fig. 4A. d-ROMs did not increase in the day of exposure (no sham effect) (Table S6; β = −0.01, p = 0.76), while BAP decreased regardless of the exposures (sham effect) (Table S6; β = −0.07, p = 0.016). After 1–3 days, both d-ROMs and BAP increased regardless of the exposures (sham effect) (Table S6; β for “1–3 days” = 0.11 with p < 0.0001, and β = 0.09 with p = 0.007, for d-ROMs and BAP, respectively). Although no effect by 28 GHz and/or heat exposures was suggested (Table S6; p = 0.18–0.96), d-ROMs (days 1–3) were plotted to visualize the multivariate-adjusted estimation, suggesting a trend toward a negative effect of 28 GHz and heat exposures (Fig. 4B).

Next, we examined the relationship between d-ROM levels (days 1–3) and ΔT or T-end. d-ROM (days 1–3) showed a negative rank correlation with T-end (Table S7; ρ = −0.33 to −0.29, p = 0.02–0.06). No such association was found for BAP levels (Table S7; ρ = −0.04 to −0.17, p = 0.24–0.89). We computed multivariate adjusted d-ROM (days 1–3) for corresponding levels of quartile ΔT or T-end (q1 to q4). As suggested in Fig. S8, d-ROM levels were relatively low in q2–q4 of rectal T-end (rectal T-end >37.2) when compared with q1 of rectal T-end (p for the difference between q1 and q2 rectal T-end = 0.056). In addition, the group with the highest tail T-end (q4) had relatively low d-ROM levels (p for the difference between q1 and q4 tail T-end = 0.059).

## 4 Discussion

In this study, young male rats were exposed to 28 GHz frequency for 40 min under normal or hot and humid ambient conditions, and stress response markers were repeatedly evaluated. There have been no prior experiments involving whole-body exposure to qMMW with dose control close to the guideline threshold, nor documented evidence regarding alterations in stress biomarkers in such settings. Because of the great difficulties in constructing an exposure system to perform 28 GHz exposure under dose control in an environment where the animals could move freely, the restraint treatment and related stress response were unavoidable. However, even with this major limitation, our study provides valuable insights into the basis for setting the appropriate threshold, because of its novelty and the accompanying dosimetry and thermophysiological assessment, which is essential for threshold discrimination. With subtracting the sham effect by the statistical models, we report that 28 GHz frequency triggered glucocorticoid synthesis as evidenced by elevated free corticosterone levels 1–3 days later. Notably, a more pronounced effect was observed in animals that experienced a change in rectal temperature of >1 °C. Additionally, the increase in urinary-free noradrenaline due to sham exposure was disturbed by both 28 GHz and heat exposure.

The effect of corticosterone was particularly robust in the animals with ΔT > 1 °C, which is consistent with, and confirms the rationale for the threshold definition in the ICNIRP’s guidelines (Fig. 1A). As a possible mechanism, we propose the involvement of heat shock proteins (HSPs), which are expressed in various organs, including the skin, and activate the transcription of the genes encoding stress proteins [30]. *Hsp90* mRNA has been reported to be induced in rat cortex after 6 h exposure to 2 GHz frequencies at 4 W/kg WBA-SAR [31]. However, our finding is novel in the following aspects. First, it is the direct evidence using a physiologically active hormone (corticosterone). Second, the latency of the response has never been reported (serum from days 1–3). Third, qMMW has not been reported in such settings, although 35 GHz reportedly induced *Hsp* in rat skin at high doses that raise core body temperature to >40 °C [32]. Since HSPs play a role in the upregulation of corticotropin-releasing hormone [33], it is possible that 28 GHz frequency-induced HSPs cause the late increase in corticosterone by promoting the synthesis of corticotropin-releasing hormone and subsequent secretion of adrenocorticotropic hormone, which promotes corticosterone production.

As evidenced by noradrenaline levels, we identified the effect of 28 GHz and heat on the inhibition of the restraint stress response. Although the underlying mechanism remains to be elucidated, we interpret this as a physiological adaptive phenomenon, i.e., heat dissipation, based on the strong inverse correlation observed between noradrenaline and tail temperature. Elevation of core body temperature above 38 °C reportedly leads to a reduction in cutaneous efferent sympathetic nerve activity, followed by the dilation of arteriovenous anastomoses (AVA) in the rat tail to discharge of superfluous thermal energy [34, 35]. Since free noradrenaline in the serum has the effect of increasing peripheral vascular resistance [36], it is possible that the decrease in noradrenaline levels played a role in AVA dilation and increased blood flow in the tail. Rodents are known to dissipate heat by applying saliva to the surface of their bodies [37–39], however, immobilization in acrylic holders should have impeded the movement. Consequently, the contribution of the tail in heat dissipation should have increased. However, the actual association between tail blood flow (area under the curve for 40 min exposure period) and noradrenaline levels (end) was not significant (ρ = −0.16, p = 0.31), suggesting that the contribution of noradrenaline to tail blood flow control was limited. Such a limited contribution is to be expected, as the increase in blood flow is also controlled by local skin heating and subsequent capillaries dilation, not by AVA [34], and because other factors such as vascular nitric oxide also contribute [40].

Unlike noradrenaline and corticosterone, no direct association with 28 GHz/heat exposure was inferred for d-ROM levels; however, d-ROM levels (days 1–3) were negatively associated with body temperature (T-end). Possible mechanisms for this phenomenon may involve 28 GHz and heat exposure elevating the body temperature, which may in turn induce HSPs. Antioxidant stress enzymes can be activated through the induction of HSPs [32, 41], leading to the suppression of the late-day increase in d-ROM levels.

Thus, we first show that whole-body exposure of rodents to 28 GHz frequency at doses up to WBA-SAR of 7.2 W/kg body weight alters their stress response. We built an exposure system with accurate dosimetry, created a valuable database including temperature, blood flow, and stress response markers, and analyzed it using efficient statistical methods. Besides the dosimetry, inclusive database, and statistical analysis, the strength of this study involves the use of a highly sensitive system that specifically measures the active (free) forms of corticosterone and catecholamines. For corticosterone, a unique and highly sensitive assay method combining solid phase extraction and LC-MS was employed; for catecholamines, a dedicated HPLC system was introduced to separate and purify derivatized compounds through two-step column switching, followed by fluorescence detection of derivatives. However, this study had some limitations. First, the use of acrylic holders for dose control to conscious rats caused significant stress and affected the results, therefore, further investigation in unrestrained rats is required. In addition, prior acclimation to the acrylic holders for half of the animals may have affected the results. We observed a higher basal corticosterone level in rats with pre-acclimation (Table S2; β = 31, p = 0.08), but did not detect any difference in increase in restraint stress (interaction p = 0.53 for acclimation × days 1–3 on corticosterone) or in the effect of 28 GHz exposure (interaction p = 0.39 for acclimation × 28 GHz × days 1–3 on corticosterone). Therefore, acclimation was unlikely to have had substantial effects on our results. Moreover, similar results were obtained from the sensitivity analysis, without adjustment for acclimation in mixed models. Additionally, the stratified analysis for each cohort showed an association in the same direction, although the significance was greatly weakened. The second limitation involves possible undetectable effects due to an insufficient number of samples and improper marker selection. For example, serum corticosterone levels may have been affected by ultradian rhythms, which have shorter cycles than circadian rhythms; this may have prevented the detection of effects that should have been evident. In the present study, efforts were made to compensate statistically for the possible reduced detection power by integrating data from the two rodent cohorts and analyzing them without stratification by the experimental condition using a multivariate-adjusted mixed model. We consider such an approach reasonable and effective in terms of scientific credibility, animal welfare, and effective use of research resources. Finally, the credibility of the findings of this study is limited because the underlying mechanism is unclear. Hence, further studies are needed to elucidate the mechanism involved.

## 5 Conclusions

To evaluate the influence of whole-body exposure to qMMW at near-threshold levels, we performed 28 GHz exposure experiments at 0, 3.7, and 7.2 W/kg body weight in young male rats restrained in acrylic holders. The 28 GHz frequency had a positive effect on serum free corticosterone levels 1–3 days after the exposure, and the effect was more pronounced in the animals with a change in rectal temperature greater than 1 °C. Both 28 GHz and heat exposure reduced free noradrenaline in the urine samples collected immediately after the exposure, suggesting a contribution to heat dissipation, a physiological adaptive phenomenon. The results of this study may provide a scientific basis for whole-body exposure thresholds for qMMW. The most likely mechanism involves the thermal effect of qMMW/heat exposure based on the association between the biomarkers and body temperatures.

## Abbreviations

AVA: arteriovenous anastomoses
BAP: biological antioxidant potential
d-ROMs: diacron reactive oxygen metabolites
5G: 5th generation wireless communication systems
HSPs: heat shock proteins
HPLC: high-performance liquid chromatography
ICNIRP: International Commission on Non-Ionizing Radiation Protection
LC: liquid chromatography
LC-MS: liquid chromatography mass spectrometry
min: minutes
qMMW: quasi-millimeter wave
WBA-SAR: whole-body average specific absorption rate

## Supplementary information

**Additional file 1:** **Figure S1.** Histogram of free corticosterone levels in rat serum. **Figure S2.** Histogram of free catecholamine levels in rat urine. **Figure S3.** Histogram of oxidative stress markers in rat serum. **Figure S4.** Correlations between time and levels of biomarkers. **Figure S5.** Multivariate-adjusted mean body temperature under 28 GHz and/or heat exposure conditions. **Figure S6.** Non-linear association between body temperatures and free corticosterone 1–3 days after the exposure treatment. **Figure S7.** Non-linear association between body temperatures and free noradrenaline at the end of exposure treatment. **Figure S8.** Non-linear association between body temperatures and d-ROM 1–3 days after the exposure treatment. **Table S1.** Spearman’s rank correlation coefficients between biomarkers and experimental conditions. **Table S2.** Partial regression coefficient (β) for fixed effects for serum corticosterone, entire cohort. **Table S3.** Spearman’s rank correlation coefficients between serum corticosterone levels on day 1 or day 3 and body temperatures of animals. **Table S4.** Partial regression coefficient (β) for fixed effects for urinary catecholamines, entire cohort. **Table S5.** Spearman’s rank correlation coefficients between catecholamine levels in urine samples collected immediately after exposure (restraint) and body temperatures of animals. **Table S6.** Partial regression coefficient (β) for fixed effects for serum oxidative stress level, entire cohort. **Table S7.** Spearman’s rank correlation coefficients between serum biomarkers and body temperature of animals.

## Declarations

### Availability of data and materials

The datasets generated and/or analyzed in the current study are available from the corresponding author upon a reasonable request.
