# Supplementary material for: Single exposure to near-threshold 5G millimeter wave modifies restraint stress responses in rats
Source: Environ Health Prev Med. 2025 May 3;30:33. doi: 10.1265/ehpm.24-00321 (PMC12062831; doi:10.1265/ehpm.24-00321)
Supplement: Supplementary file 1 — Additional file 1: Figure S1. Histogram of free corticosterone levels in rat serum. Figure S2. Histogram of free catecholamine levels in rat urine. Figure S3. Histogram of oxidative stress markers in rat serum. Figure S4. Correlations between time and levels of biomarkers. Figure S5. Multivariate-adjusted mean body temperature under 28 GHz and/or heat exposure conditions. Figure S6. Non-linear association between body temperatures and free corticosterone 1–3 days after the exposure treatment. Figure S7. Non-linear association between body temperatures and free noradrenaline at the end of exposure treatment. Figure S8. Non-linear association between body temperatures and d-ROM 1–3 days after the exposure treatment. Table S1. Spearman’s rank correlation coefficients between biomarkers and experimental conditions. Table S2. Partial regression coefficient (β) for fixed effects for serum corticosterone, entire cohort. Table S3. Spearman’s rank correlation coefficients between serum corticosterone levels on day 1 or day 3 and body temperatures of animals. Table S4. Partial regression coefficient (β) for fixed effects for urinary catecholamines, entire cohort. Table S5. Spearman’s rank correlation coefficients between catecholamine levels in urine samples collected immediately after exposure (restraint) and body temperatures of animals. Table S6. Partial regression coefficient (β) for fixed effects for serum oxidative stress level, entire cohort. Table S7. Spearman’s rank correlation coefficients between serum biomarkers and body temperature of animals. [file ehpm-30-033-s001.docx]

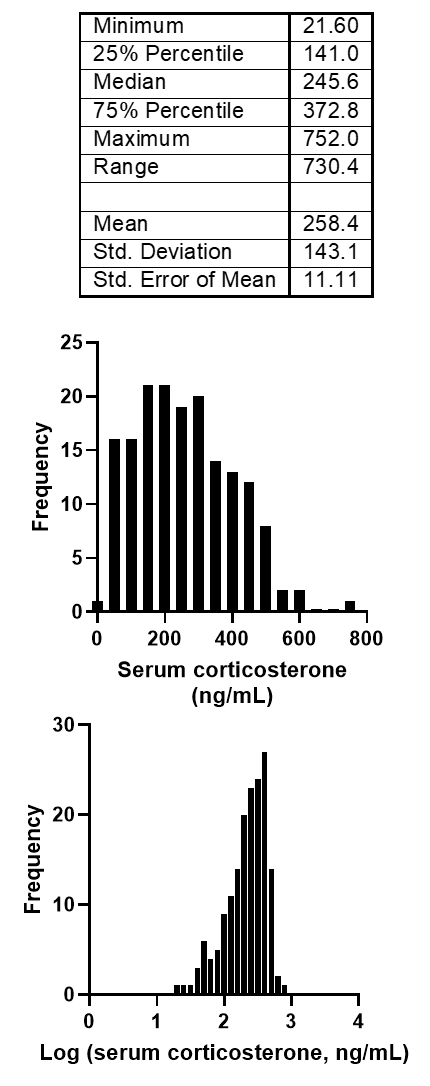


**Figure S1. Histogram of free corticosterone levels in rat serum.**

The distribution of serum corticosterone levels was not normalized by logarithmic transformation.


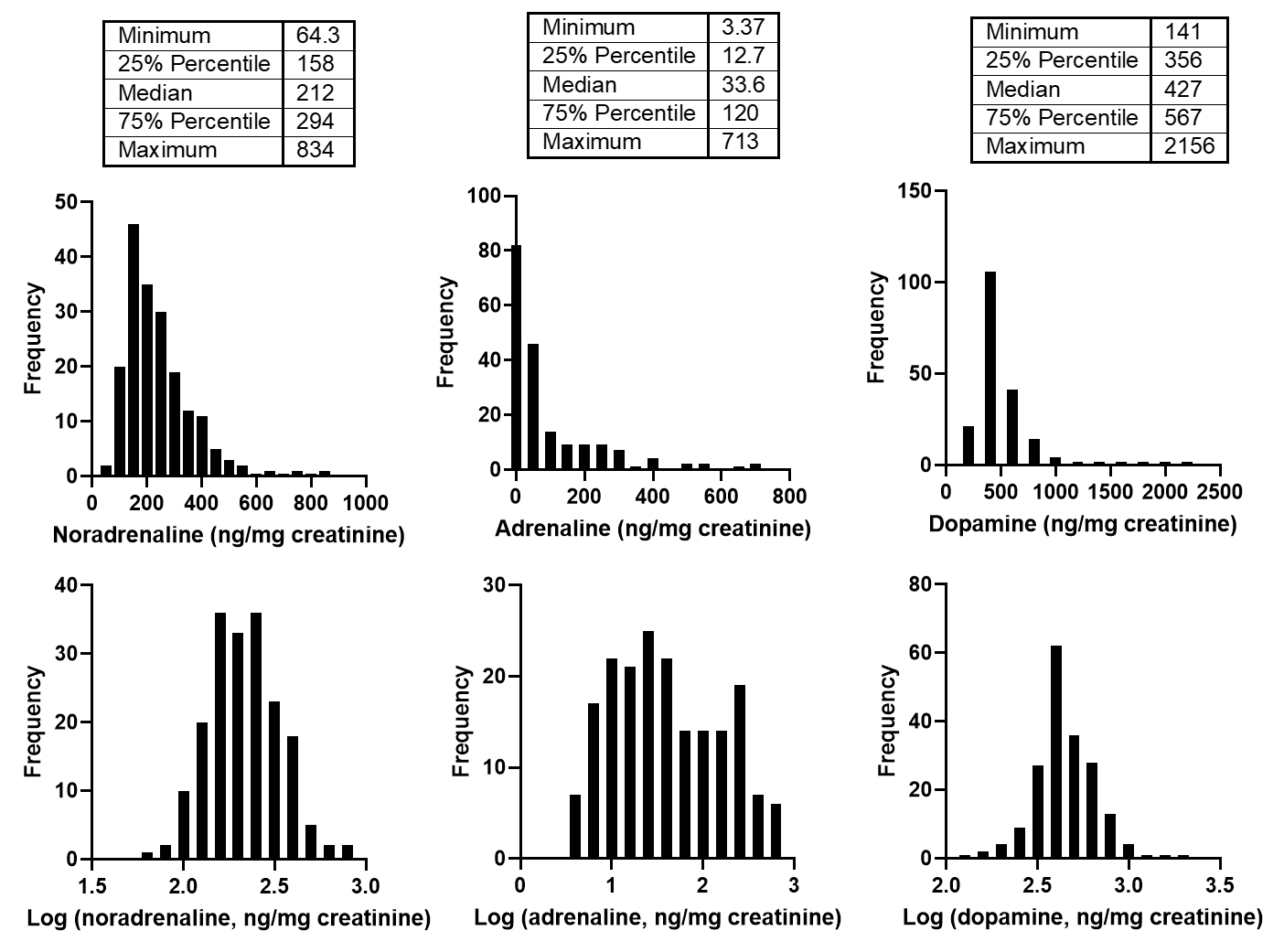


**Figure S2. Histogram of free catecholamine levels in rat urine.**

The urinary catecholamine levels were subjected to logarithmic transformation to approximate normal distributions.


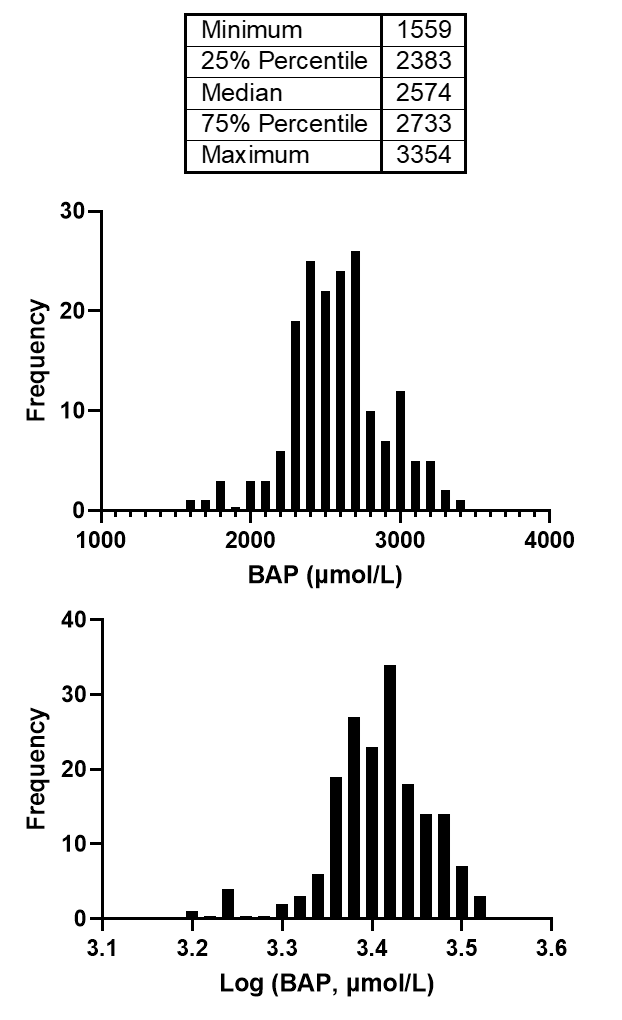

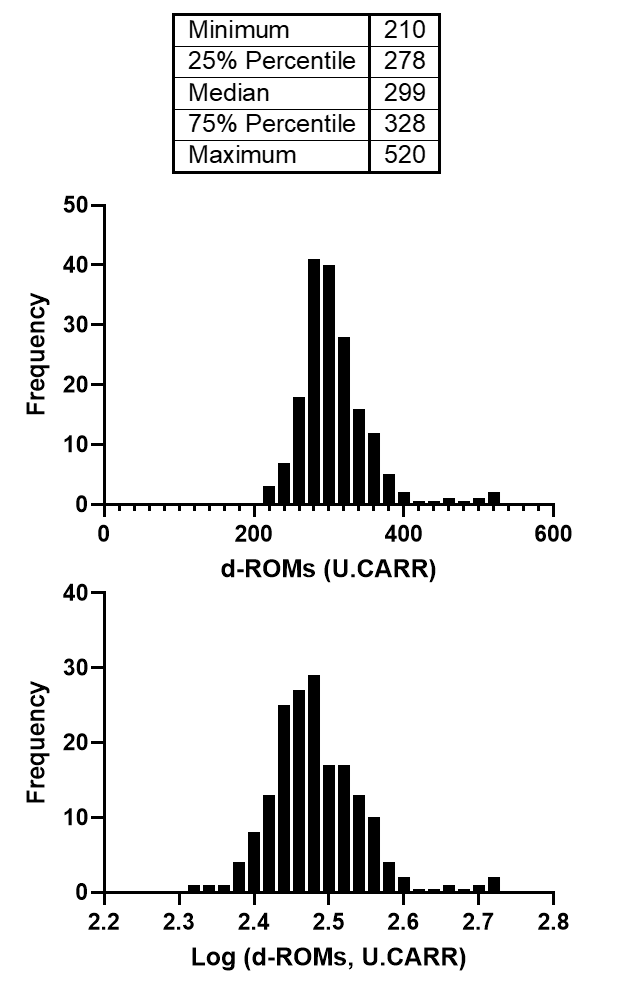


**Figure S3. Histogram of oxidative stress markers in rat serum.**

The d-ROMs and BAPs were subjected to logarithmic transformation to approximate normal distributions.

| **Table S1. Spearman's rank correlation coefficients between biomarkers and experimental conditions.** | | |
| --- | --- | --- |
|  | vs. acclimation with adjustment of sampling time | vs. sampling time with adjustment of acclimation |
| Urinary noradrenaline | −0.140 | 0.174 |
| N = 50 | p = 0.3375 | p = 0.2325 |
| Urinary adrenaline | 0.022 | 0.125 |
| N = 50 | p = 0.8785 | p = 0.3927 |
| Urinary dopamine | −**0.525** | 0.202 |
| N = 50 | **p = 0.0001** | p = 0.1644 |
| Serum corticosterone | 0.101 | 0.102 |
| N = 58 | p = 0.4541 | p = 0.4497 |
| d-ROMs | −0.107 | −0.032 |
| N = 57 | p = 0.4312 | p = 0.8159 |
| BAP | **0.279** | 0.007 |
| N = 57 | **p = 0.0372** | p = 0.9583 |


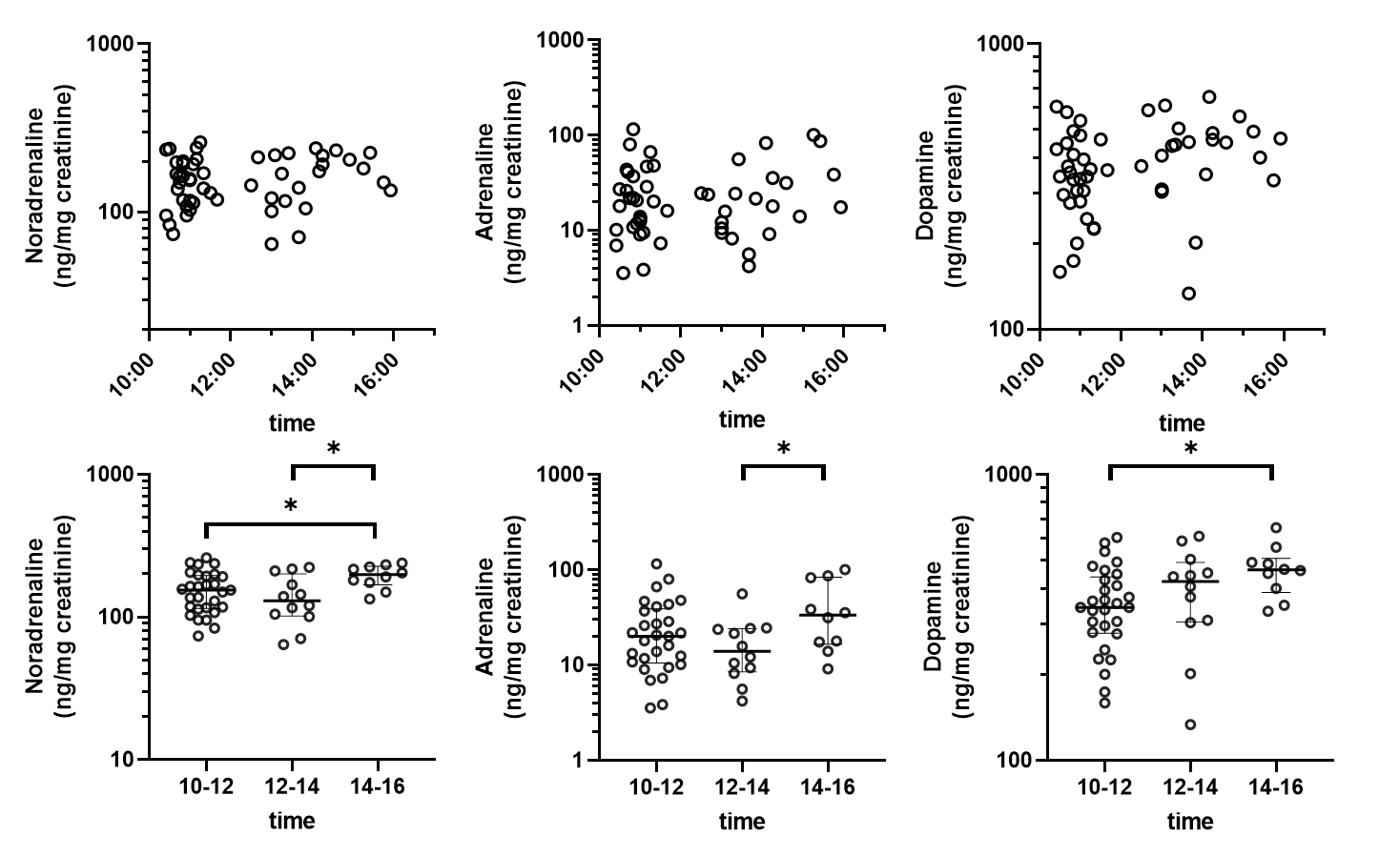


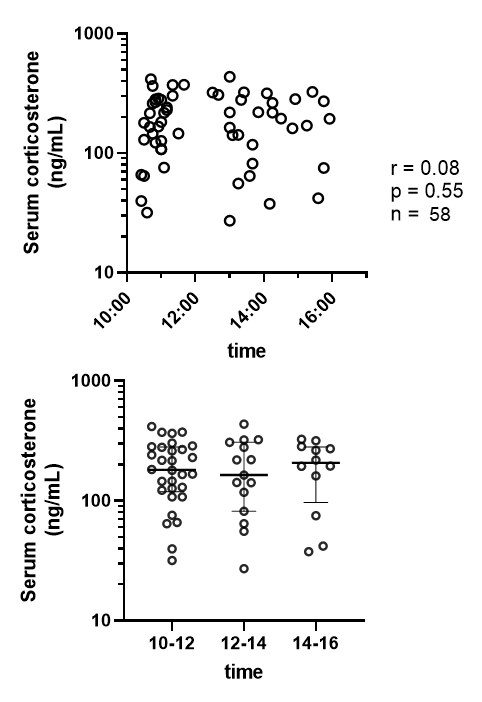


**Figure S4. Correlations between time and levels of biomarkers.**

Urine and blood specimens were collected under anesthesia prior to the day of 28 GHz exposure experiment. r, Spearman rank correlation coefficient; n, number of observations; *, *p* <0.05, **, *p* <0.01 by Mann–Whitney U test.


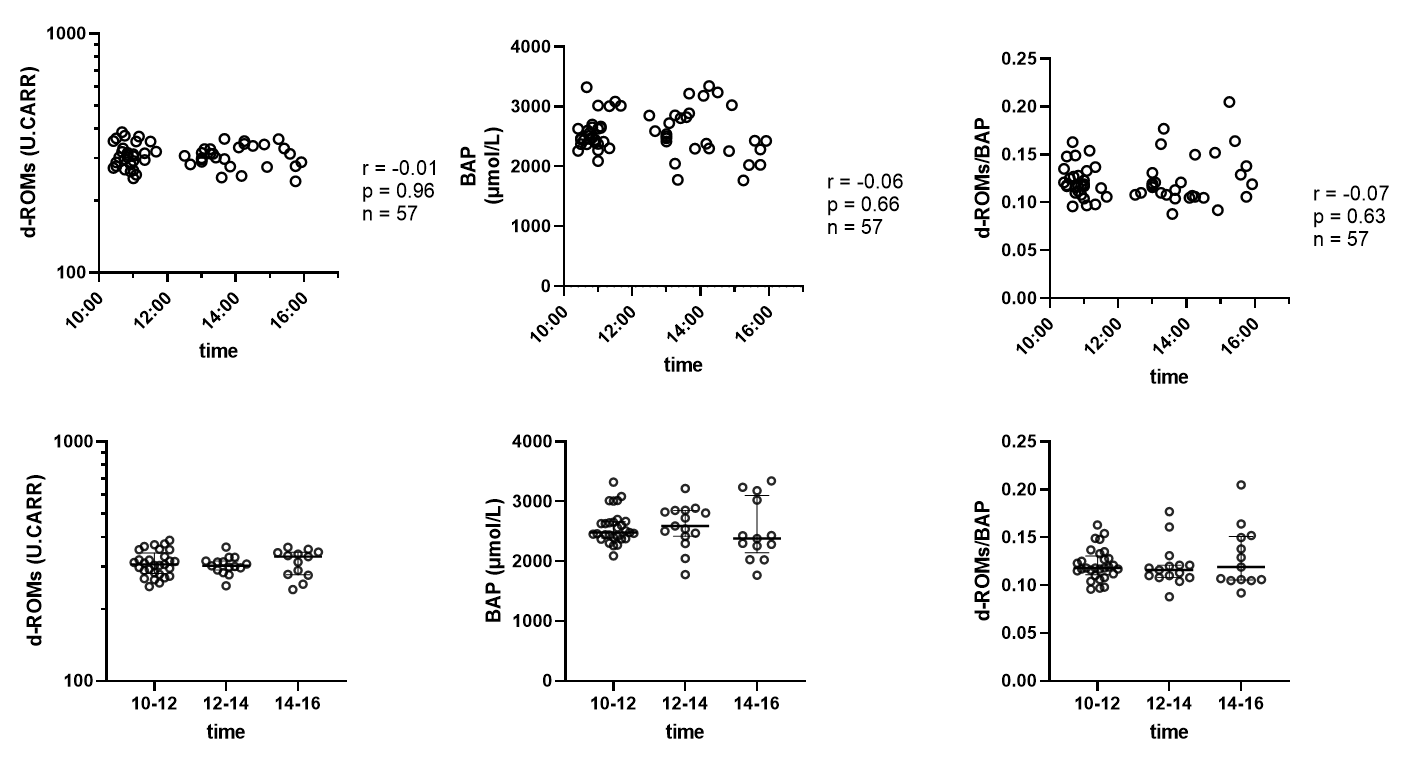


**Figure S4. Continued.**

**Figure S5. Multivariate-adjusted mean body temperature under 28 GHz and/or heat exposure conditions.**

The estimated changes in body temperatures (least squares mean and standard error of the mean) were computed using an integrated database **(A)**. The temperatures at the end of the exposure treatment were estimated using a similar method to A **(B)**. To account for differences in experimental conditions, multivariate-adjusted estimation was performed using a mixed model with fixed effects of acclimation procedures performed before exposure experiments, the time of sample collection (before/after 14:00), and the random effect of the time of rat procurement (time of the experiment). Red circles indicate heat exposure at a temperature of 31.5 °C and humidity of 70%. WBA-SAR: whole-body average specific absorption rate.

| **Table S2. Partial regression coefficient (β) for fixed effects for serum corticosterone, entire cohort.** | | |
| --- | --- | --- |
| Fixed effects | β | p-value |
| Intercept | 164.19 |  |
| Acclimation to animal holder prior to 28 GHz exposure (restraint) | 31.04 | 0.0795 |
| Time of sample collection (14:00) | 6.02 | 0.6721 |
|  |  |  |
| Previous day (sham effect) | 17.10 | 0.3292 |
| Immediately before the exposure (sham effect) | (reference) |  |
| **End of the exposure period** (sham effect) | **237.68** | **<0.0001** |
| 1–3 h after the end of the exposure (sham effect) | 27.92 | 0.2862 |
| **1–3 days after the exposure** (sham effect) | **86.33** | **0.0008** |
|  |  |  |
| 28 GHz exposure (one order increase) × end of the exposure | 27.36 | 0.1666 |
| 28 GHz exposure (one order increase) × 1–3 h after the end of the exposure | −18.50 | 0.3534 |
| **28 GHz exposure (one order increase) × 1**–**3 days after the exposure** | **55.05** | **0.0049** |
|  |  |  |
| Heat × end of the exposure | 9.47 | 0.7696 |
| Heat × 1–3 h after the end of the exposure | −12.97 | 0.6843 |
| Heat × 1–3 days after the exposure | 26.67 | 0.3956 |
| Due to the small number of specimens on day 1 and day 3, one category was assigned as day 1–3. The mixed model includes fixed effects as follows: time of sample collection (before/after 14:00), time course (previous day, start, end, 1–3 h, day1–3), interactive term of 28 GHz intensity (0, 3.7, and 7.2 W/kg as 3 ordinal variables) and time course (end, 1–3 h, day 1–3), and interactive term of the environment (normal/heat), 28 GHz intensity, and time course (end, 1–3 h, day 1–3). | | |

| **Table S3. Spearman's rank correlation coefficients between serum corticosterone levels on day 1 or day 3 and body temperatures of animals.** | | | |
| --- | --- | --- | --- |
|  | Rectal | Dermal | Tail |
| Serum corticosterone on day 1 or day 3 | vs temperature at the end (T-end) | | |
|  | 0.27 | 0.25 | 0.20 |
|  | p = 0.0602 | p = 0.1015 | p = 0.1696 |
|  | vs change in temperature (after – before) (ΔT) | | |
|  | **0.37** | **0.39** | 0.28 |
|  | **p = 0.0099** | **p = 0.009** | p = 0.0584 |
| The rank correlations were computed with adjustment of acclimation, time of sample collection (before/after 14:00), and days after the exposure. | | | |


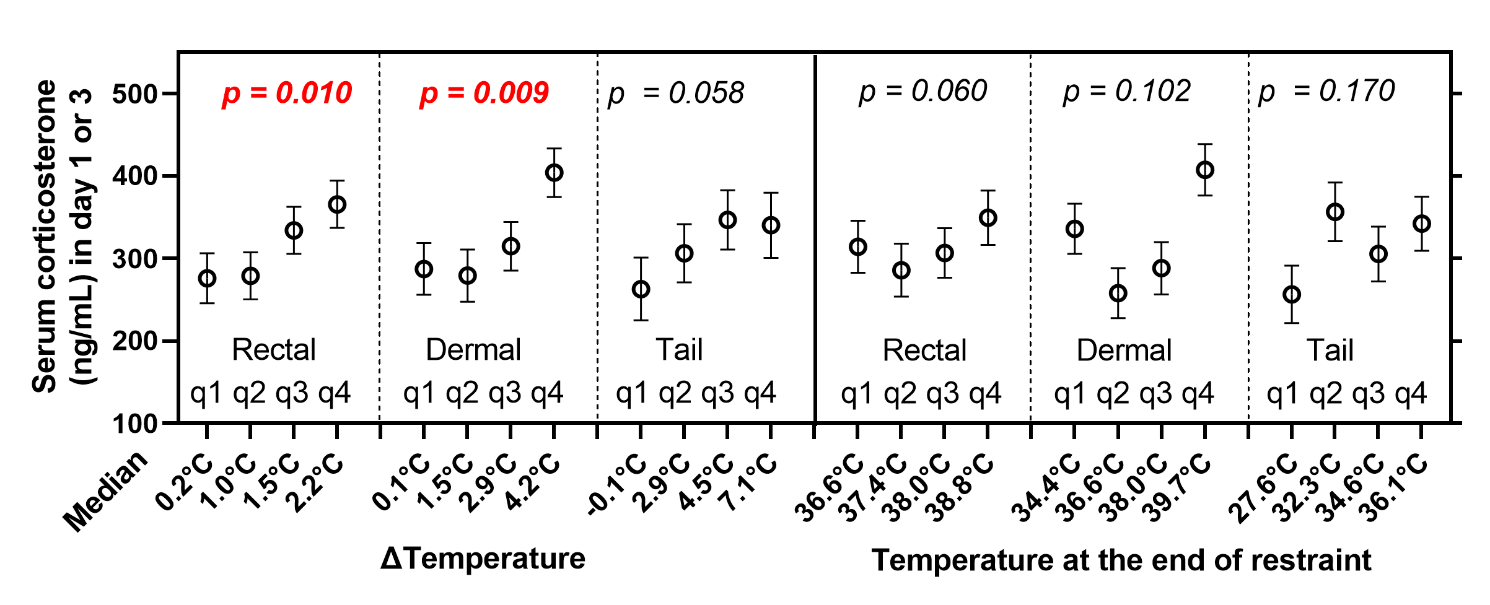


**Figure S6. Non-linear association between body temperatures and free corticosterone 1–3 days after the exposure treatment.**

To depict the non-linear relationships between body temperatures and serum-free corticosterone, observations were divided into quadrants by body temperature. The multi-adjusted means and SEMs for each category of body temperatures were plotted. Least squares means and standard errors based on quartiles of Δ temperature or the temperature at the end of exposure treatment (N = 51, 47, and 50 for rectal, dermal, and tail temperatures, respectively) were computed using a mixed model with fixed effects of acclimation procedures performed before exposure experiments, the time of sample collection (before/after 14:00), days after exposure treatment (1 or 3), and the random effect of the time of rat procurement (time of the experiment). ‘Heat’ indicates exposure to a hot and humid environment at the experimental temperature of 31.5 °C and humidity of 70%. The p-values are for Spearman's rank correlation coefficients computed with adjustment for acclimation, time of sample collection (before/after 14:00), and days after exposure treatment (1 or 3) (see Table S3).

| **Table S4. Partial regression coefficient (β) for fixed effects for urinary catecholamines, entire cohort.** | | |
| --- | --- | --- |
| Fixed effects | β | p-value |
| Dependent variable = Log (noradrenaline, ng/mg creatinine) |  |  |
| Intercept | 5.10 |  |
| Acclimation to animal holder prior to 28 GHz exposure (restraint) | −0.03 | 0.5686 |
| Time of sample collection (14:00) | 0.08 | 0.1788 |
|  |  |  |
| Previous day (sham effect) | −0.06 | 0.2739 |
| Immediately before the exposure (sham effect) | (reference) |  |
| **End of the exposure**  (sham effect) | **0.91** | **<.0001** |
| **1–3 h after the end of the exposure** (sham effect) | **0.44** | **<.0001** |
|  |  |  |
| **28 GHz exposure (one order increase) × end of the exposure** | −**0.14** | **0.0233** |
| 28 GHz exposure (one order increase) × 1−3 h after the end of the exposure | 0.02 | 0.7895 |
| **Heat× end of the exposure** | −**0.38** | **0.0009** |
| Heat× 1–3 h after the end of the exposure | −0.07 | 0.535 |
| Dependent variable = Log (adrenaline, ng/mg creatinine) |  |  |
| Intercept | 2.51 |  |
| Acclimation to animal holder prior to 28 GHz exposure (restraint) | −0.06 | 0.6483 |
| Time of sample collection (14:00) | −0.16 | 0.2474 |
|  |  |  |
| **Previous day** (sham effect) | **0.58** | **<.0001** |
| Immediately before the exposure (sham effect) | (reference) |  |
| **End of the exposure** (sham effect) | **3.16** | **<.0001** |
| **1–3 h after the end of the exposure** (sham effect) | **1.36** | **<.0001** |
|  |  |  |
| 28 GHz exposure (one order increase) × end of the exposure | −0.05 | 0.7333 |
| 28 GHz exposure (one order increase) × 1−3 h after the end of the exposure | −0.05 | 0.7767 |
| Heat× end of the exposure | −0.43 | 0.115 |
| Heat× 1–3 h after the end of the exposure | −0.02 | 0.9329 |
| Dependent variable = Log (dopamine, ng/mg creatinine) |  |  |
| Intercept | 6.10 |  |
| **Acclimation to animal holder prior to 28 GHz exposure (restraint)** | **−0.23** | **0.0105** |
| **Time of sample collection (14:00)** | **0.18** | **0.0021** |
|  |  |  |
| Previous day (sham effect) | −0.04 | 0.4331 |
| Immediately before the exposure (sham effect) | (reference) |  |
| **End of the exposure period** (sham effect) | **0.41** | **<.0001** |
| 1–3 h after the end of the exposure (sham effect) | −0.01 | 0.9485 |
|  |  |  |
| 28 GHz exposure (one order increase) × end of the exposure | 0.01 | 0.8762 |
| 28 GHz exposure (one order increase) × 1–3 h after the end of the exposure | 0.01 | 0.8971 |
| Heat× end of the exposure | −0.04 | 0.7172 |
| Heat× 1–3 h after the end of the exposure | −0.08 | 0.5103 |
| The mixed model includes fixed effects as follows, time of sample collection (before/after 2 PM), time course (previous day, start, end, and 1–3 h), interactive term of 28 GHz intensity (0, 3.7, and 7.2 W/kg as 3 ordinal variables) and time course (end, 1–3 h), and interactive term of the environment (normal/heat), 28 GHz intensity, and time course (end, 1–3 h). | | |

| **Table S5. Spearman's rank correlation coefficients between catecholamine levels in urine samples collected immediately after exposure (restraint) and body temperatures of animals.** | | | |
| --- | --- | --- | --- |
|  | Rectal | Dermal | Tail |
|  | vs temperature at the end (T-end) | | |
| Noradrenaline | −0.29 | −0.22 | −**0.46** |
|  | p = 0.0707 | p = 0.1962 | **p = 0.0037** |
| Adrenaline | −0.19 | −0.28 | −0.28 |
|  | p = 0.2463 | p = 0.0938 | p = 0.0928 |
| Dopamine | 0.06 | 0.05 | −0.13 |
|  | p = 0.7221 | p = 0.7885 | p = 0.427 |
|  | vs change in temperature (after – before) (ΔT) | | |
| Noradrenaline | −0.09 | −0.12 | −0.27 |
|  | p = 0.5800 | p = 0.4947 | p = 0.0967 |
| Adrenaline | −0.05 | −0.09 | −0.20 |
|  | p = 0.7372 | p = 0.5917 | p = 0.2189 |
| Dopamine | 0.26 | 0.13 | −0.01 |
|  | p = 0.1008 | p = 0.4466 | p = 0.9594 |
| The rank correlations were computed with adjustment of acclimation and time of sample collection (before/after 14:00). | | | |


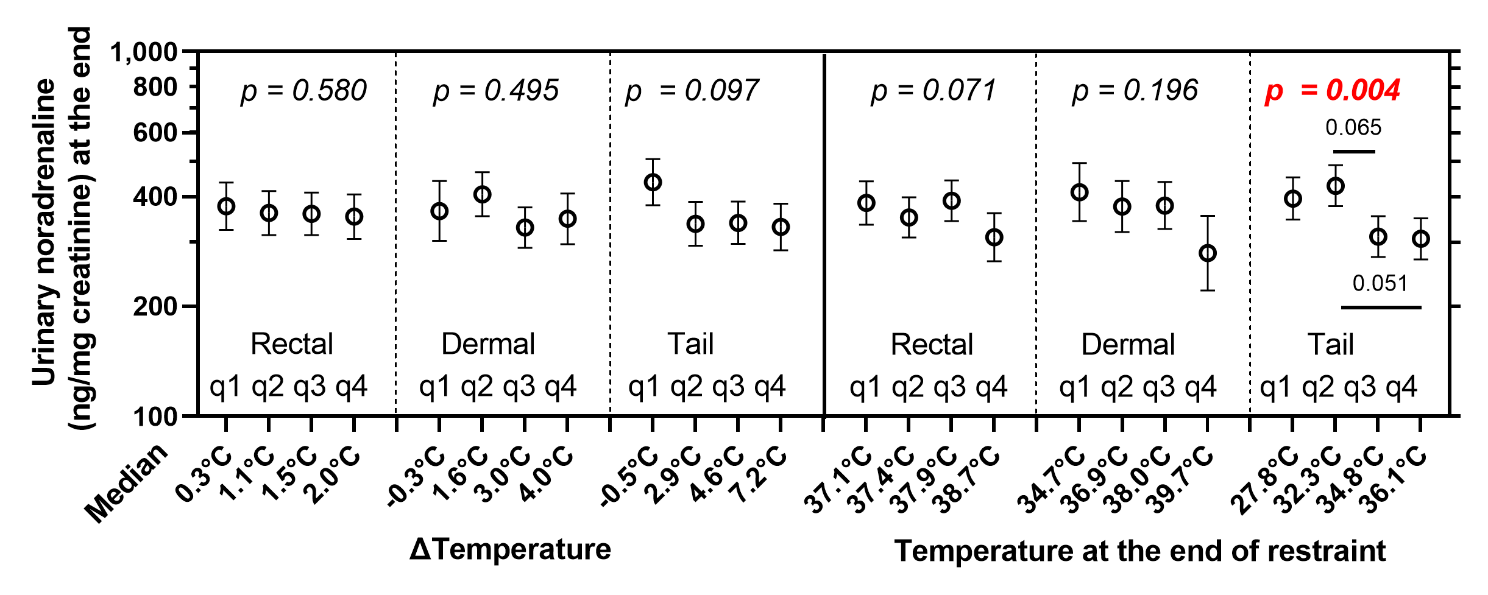


**Figure S7. Non-linear association between body temperatures and free noradrenaline at the end of exposure treatment.**

To depict the non-linear relationships between body temperatures and urinary-free noradrenaline, observations were divided into quadrants by body temperature. The multi-adjusted geometric means and SEMs for each category of body temperatures were plotted. Least squares geometric means and standard errors based on quartiles of Δ temperature or the temperature at the end of exposure treatment (N = 45, 42, and 42 for rectal, dermal, and tail temperatures, respectively) were computed using a mixed model with fixed effects of acclimation procedures performed before exposure experiments, the time of sample collection (before/after 14:00), and the random effect of the time of rat procurement (time of the experiment). ‘Heat’ indicates exposure to a hot and humid environment at the experimental temperature of 31.5 °C and humidity of 70%. The p-values are for Spearman's rank correlation coefficients computed with adjustment for acclimation and time of sample collection (before/after 14:00) (see Table S5).

| **Table S6. Partial regression coefficient (β) for fixed effects for serum oxidative stress level, entire cohort.** | | |  |
| --- | --- | --- | --- |
| Fixed effects | β | *p*-value |  |
| Dependent variable = Log (d-ROMs, U.CARR) |  |  |  |
| Intercept | 5.69 |  |  |
| Acclimation to animal holder prior to 28 GHz exposure (restraint) | 0.03 | 0.4202 |  |
| Time of sample collection (14:00) | 0.01 | 0.4936 |  |
|  |  |  |  |
| Previous day (sham effect) | −0.006 | 0.7106 |  |
| Immediately before the exposure (sham effect) | (reference) |  |  |
| End of the exposure period (sham effect) | −0.008 | 0.7595 |  |
| 1–3 h after the end of the exposure (sham effect) | −0.023 | 0.3767 |  |
| **1–3 days after the exposure** (sham effect) | **0.107** | **<0.0001** |  |
|  |  |  |  |
| 28 GHz exposure (one order increase) × end of the exposure | −0.015 | 0.4565 |  |
| 28 GHz exposure (one order increase) × 1–3 h after the end of the exposure | −0.001 | 0.9685 |  |
| 28 GHz exposure (one order increase) × 1–3 days after the exposure | −0.015 | 0.4639 |  |
|  |  |  |  |
| Heat × end of the exposure | 0.027 | 0.4251 |  |
| Heat × 1–3 h after the end of the exposure | −0.027 | 0.4168 |  |
| Heat × 1–3 days after the exposure | −0.044 | 0.1853 |  |
| Dependent variable = Log (BAP, μmol/L) |  |  |  |
| Intercept | 7.85 |  |  |
| Acclimation to animal holder prior to 28 GHz exposure (restraint) | 0.03 | 0.2824 |  |
| Time of sample collection (14:00) | 0.01 | 0.4381 |  |
|  |  |  |  |
| Previous day (sham effect) | −0.013 | 0.5472 |  |
| Immediately before the exposure (sham effect) | (reference) |  |  |
| **End of the exposure period** (sham effect) | **−0.073** | **0.0157** |  |
| 1–3 h after the end of the exposure (sham effect) | −0.002 | 0.9608 |  |
| **1–3 days after the exposure** (sham effect) | **0.085** | **0.0068** |  |
|  |  |  |  |
| 28 GHz exposure (one order increase) × end of the exposure | 0.008 | 0.7272 |  |
| 28 GHz exposure (one order increase) × 1–3 h after the end of the exposure | 0.030 | 0.2173 |  |
| 28 GHz exposure (one order increase) × 1–3 days after the exposure | 0.033 | 0.1760 |  |
|  |  |  |  |
| Heat × end of the exposure | 0.036 | 0.3397 |  |
| Heat × 1–3 h after the end of the exposure | 0.005 | 0.8967 |  |
| Heat × 1–3 days after the exposure | −0.045 | 0.2281 |  |
| Due to the small number of specimens on day 1 and day 3, one category was assigned as day 1–3. The mixed model includes fixed effects as follows, time of sample collection (before/after 14:00), time course (previous day, start, end, 1–3 h, day1–3), interactive term of 28 GHz intensity (0, 3.7, and 7.2 W/kg as 3 ordinal variables) and time course (end, 1–3 h, day 1–3), and interactive term of the environment (normal/heat), 28 GHz intensity, and time course (end, 1–3 h, day 1–3). | | |  |
|  |  |  |  |

| **Table S7. Spearman's rank correlation coefficients between serum biomarkers and body temperature of animals.** | | | |
| --- | --- | --- | --- |
|  | Rectal | Dermal | Tail |
|  | vs temperature at the end (T-end) | | |
| d-ROMs on day 1 or day 3 | **−0.29** | −0.29 | **−0.33** |
|  | **p = 0.04** | p = 0.06 | **p = 0.02** |
| BAP on day 1 or day 3 | −0.04 | 0.05 | −0.02 |
|  | p = 0.78 | p = 0.75 | p = 0.89 |
|  | vs change in temperature (after – before) (ΔT) | | |
| d-ROMs on day 1 or day 3 | −0.14 | −0.17 | −0.17 |
|  | p = 0.34 | p = 0.27 | p = 0.26 |
| BAP on day 1 or day 3 | 0.17 | 0.16 | 0.05 |
|  | p = 0.24 | p = 0.31 | p = 0.74 |
| The rank correlations were computed with adjustment of acclimation, time of sample collection (before/after 14:00), and days after the exposure. | | | |


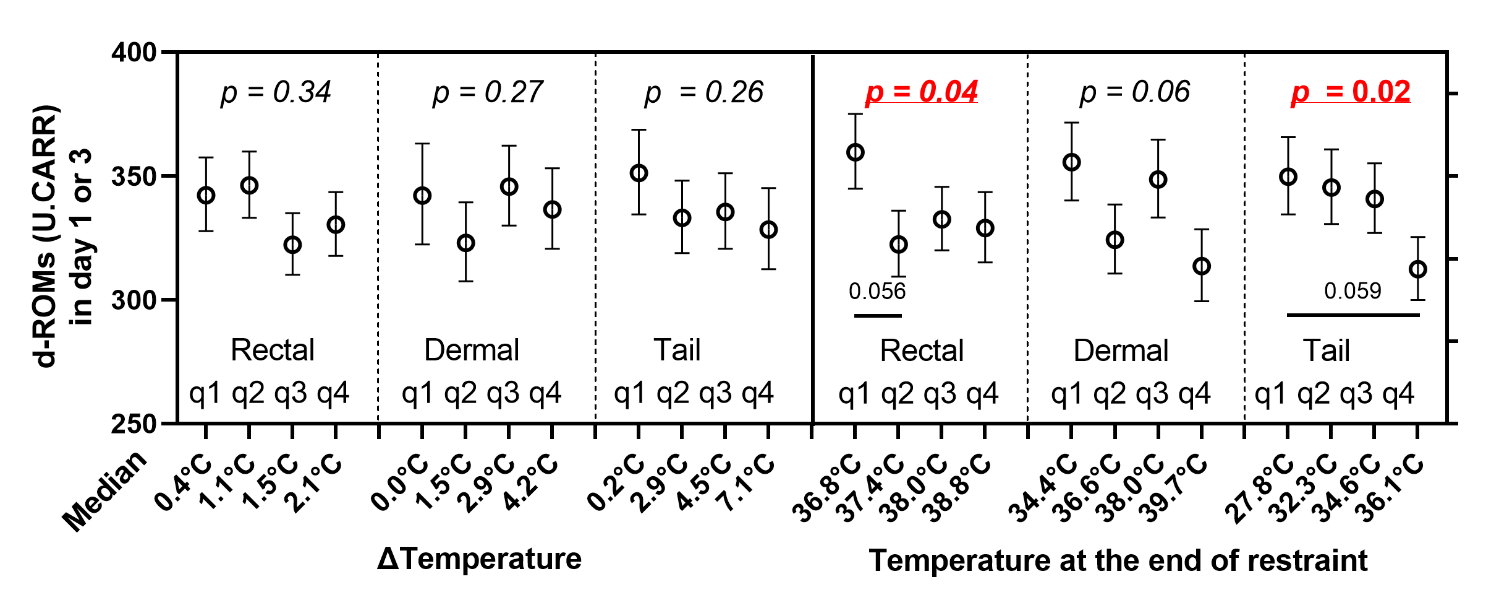


**Figure S8. Non-linear association between body temperatures and d-ROM 1–3 days after the exposure treatment.**

To depict the non-linear relationships between body temperatures and d-ROMs, observations were divided into quadrants by body temperature. The multi-adjusted geometric means and SEMs for each category of body temperatures were plotted. Least squares geometric means and standard errors based on quartiles of Δ temperature or the temperature at the end of exposure treatment (N = 50, 46, and 49 for rectal, dermal, and tail temperatures, respectively) were computed using a mixed model with fixed effects of acclimation procedures performed before exposure experiments, the time of sample collection (before/after 14:00), days after exposure treatment (1 or 3), and the random effect of the time of rat procurement (time of the experiment). ‘Heat’ indicates exposure to a hot and humid environment at the experimental temperature of 31.5 °C and humidity of 70%. The p-values are for Spearman's rank correlation coefficients computed with adjustment for acclimation, time of sample collection (before/after 14:00), and days after exposure treatment (1 or 3) (see Table S7).
